# Does additional resection of a positive microscopic ductal margin benefit patients with perihilar cholangiocarcinoma: A systematic review and meta-analysis

**DOI:** 10.1371/journal.pone.0232590

**Published:** 2020-05-07

**Authors:** Qiao Ke, Yuqing Chen, Qizhen Huang, Nanping Lin, Lei Wang, Jingfeng Liu

**Affiliations:** 1 Department of Hepatopancreatobiliary Surgery, Mengchao Hepatobiliary Hospital of Fujian Medical University, Fuzhou, Fujian, China; 2 The Graduate School of Fujian Medical University, Fuzhou, Fujian, China; 3 Department of Radiation Oncology, Mengchao Hepatobiliary Hospital of Fujian Medical University, Fuzhou, Fujian, China; 4 Department of Radiation Oncology, Fujian Medical University Cancer Hospital, Fujian Cancer Hospital, Fuzhou, China; Texas A&M University, UNITED STATES

## Abstract

**Background:**

The incidence of a positive microscopic ductal margin (R1) after surgical resection for perihilar cholangiocarcinoma (pCCA) remains high, but the beneficial of additional resection has not been confirmed by any meta-analysis and randomized clinical trials (RCT), which also increased the risk of morbidity and mortality. Hence, a systematic review is warranted to evaluate the clinical value of additional resection of intraoperative R1 for pCCA.

**Methods:**

Eligible studies were searched by PubMed, MedLine, Embase, the Cochrane Library, Web of Science, from Jan.1^st^ 2000 to Nov.30^th^ 2019, evaluating the 1-, 3-, and 5-year overall survival (OS) rates of additional resection of intraoperative pathologic R1 for pCCA. Odds ratio (OR) with 95% confidence interval (CI) was used to determine the effect size by a randomized-effect model.

**Results:**

Eight studies were enrolled in this meta-analysis, including 179 patients in the secondary R0 group, 843 patients in the primary R0 group and 253 patients in the R1 group. The pooled OR for the 1-, 3-, and 5-year OS rate between secondary R0 group and primary R0 group were 1.03(95%CI 0.64~1.67, P = 0.90), 0.92(95%CI 0.52~1.64, P = 0.78), and 0.83(95%CI 0.37~1.84, P = 0.65), respectively. The pooled OR for the 1-, 3-, and 5-year OS rate between secondary R0 group and R1 group were 2.14(95%CI 1.31~3.50, P = 0.002), 2.58(95%CI 1.28~5.21, P = 0.008), and 3.54(95%CI 1.67~7.50, P = 0.001), respectively. However, subgroup analysis of the West showed that the pooled OR for the 1-, and 3-year OS rate between secondary R0 group and R1 group were 2.05(95%CI 0.95~4.41, P = 0.07), 1.91(95%CI 0.96~3.81, P = 0.07), respectively.

**Conclusion:**

With the current data, additional resection should be recommended in selected patients with intraoperative R1, but the conclusion is needed further validation.

## Introduction

The incidence of perihilar cholangiocarcinoma (pCCA) is increasing stably [1], but the prognosis is generally poor [2, 3]. Surgical resection is the only potential way to achieve a long survival [3, 4], although only 20% patients are operable at diagnosis [5, 6]. However, the 5-year survival rates were reported to be 15~40% [7–9], and the incidence of recurrence within two year was reported to be as high as 80% [10]. Positive bile duct margin is one of the most important factors for poor prognosis of pCCA after resection [11–13], and the incidence of a positive microscopic ductal margin (R1) ranged from 10~72% [11, 13, 14]. Hence, additional resection is necessary to achieve a margin-negative (R0) resection and improved prognosis once R1 was confirmed by intraoperative frozen pathology.

However, additional resection of pCCA typically increased the risk of perioperative morbidity and mortality [14–16]. In addition, secondary R0 resection is hard to achieve in selected patients with more advanced disease and concurrent major vascular invasion [17, 18]. What’s more, it remains controversial whether patients with pCCA could be benefited from additional resection [13, 14, 17, 19], and to the best of our knowledge, no meta-analysis or randomized clinical trials (RCT) have been published on this issue. Hence, a meta-analysis was warranted to evaluate the clinical value of additional resection of intraoperative R1 for pCCA.

## Material and method

This study was based on published studies and the informed consent of the patients and the ethical approval were not required. This meta-analysis was conducted according to the preferred Reporting Items for Systematic Reviews and Meta-Analyses (PRISMA).

### Literature search

A comprehensive search on the existing published medical literature was conducted by two independent researchers to investigate the outcomes of additional resection of intraoperative R1 for pCCA. English electronic databases such as PubMed, MedLine, Embase, the Cochrane Library, Web of Science were used to search the literature from Jan.1^st^ 2000 to Nov.30^th^ 2019. Key words were as follows: (“hilar cholangiocarcinoma” or “perihilar cholangiocarcinoma” or “Klatskin’s tumor” or “HCCA” or “pCCA”) AND (“additional resection” or “extensive resection” or “re-resection”) AND (“margin”). The references of the included studies, relevant meta-analyses, reviews and guidelines were manually screened to look for potentially eligible studies.

### Selection criteria

Inclusion criteria: i) patients with pCCA; ii) additional resection were conducted once R1 was confirmed by intraoperative frozen pathology; iii) comparison between secondary R0 and primary R0 or R1; iv) outcomes including overall survival (OS) rate; v) either randomized controlled trial (RCT) or retrospective studies.

Exclusion criteria: i) patients including non-pCCA; ii) data on the OS rates was not available; iii) grouping information was blur; ⅳ) studies based on overlapping cohorts deriving from the same center.

### Intervention

Hepatectomy with en-bloc total caudate lobe resection, and regional lymphadenectomy along with (+/−) vascular resection and reconstruction was conducted as a standard procedure for pCCA [4, 20], but the detailed procedure was different from each center [4, 20].

Both distal bile duct margin (DM) and proximal bile duct margin (PM) were collected to conduct an intraoperative frozen-section examination, and were evaluated by at least two pathologists within 30 min [21]. Of note, R1 was defined as microscopical positive ductal margin, including invasive carcinoma (R-inv) and carcinoma in situ (R-cis) [19].

Additional resection was performed when either DM or PM was positive. Additional resection typically involved extended hepatectomy and/or extirpation of the biliary tree, but the detailed procedure was different from each center [13, 14, 17, 18].

### Data extraction

Data such as the author’s first name, year of publication, study methods, patient’s characteristic, interventions, and outcomes were extracted and assessed by two independent investigators with predefined forms such as baseline characteristics and outcomes from each study. The odd ratios (ORs) of 1-, 3-, and 5-year OS were extracted directedly from the original data or extracted from the Kaplan-Meier curves according to the methods described in detail by Tierney et al [22]. and Parmar et al [23]. In case of disagreement, a third investigator intervened for a conclusion.

### Quality assessment

The quality of non-randomized studies was assessed by the modified Newcastle-Ottawa Scale (NOS) [24], and more than 7 stars were defined as high quality, 4~6 star as medium quality, and <4 stars as low quality.

### Statistical analysis

The meta-analysis was registered at http://www.crd.york.ac.uk/PROSPERO/ (Review registry 133971) and was performed using RevMan Version 5.3. ORs and 95%CIs were used to evaluate the 1-, 3-, and 5-year OS rates between secondary R0 and primary R0 or R1. Considering the inherent heterogeneity among the included studies, the pooled ORs for 1-, 3-, and 5-year OS rates were evaluated by the random-effects model [25]. But in the subgroup analysis, to choose whether random-effects or fixed-effects model was determined by heterogeneity test. The heterogeneity was assessed by the χ^2^ test and I^2^ statistics; P < 0.10 or I^2^ > 50% were considered as significant heterogeneity. When the hypothesis of homogeneity was rejected, the fixed-effects model was used to estimate the case with homogeneity, and the random-effects model was used for the cases with significant heterogeneity [26]. Sensitivity analysis was conducted as follows: one study at a time was removed and the remained were re-analyzed to determine whether the results could be affected significantly by single study [27]. Begg’s and Egger’s tests were used to evaluate publication bias using Stata 14.

## Results

### Base characteristic of the included studies

**Fig 1:**
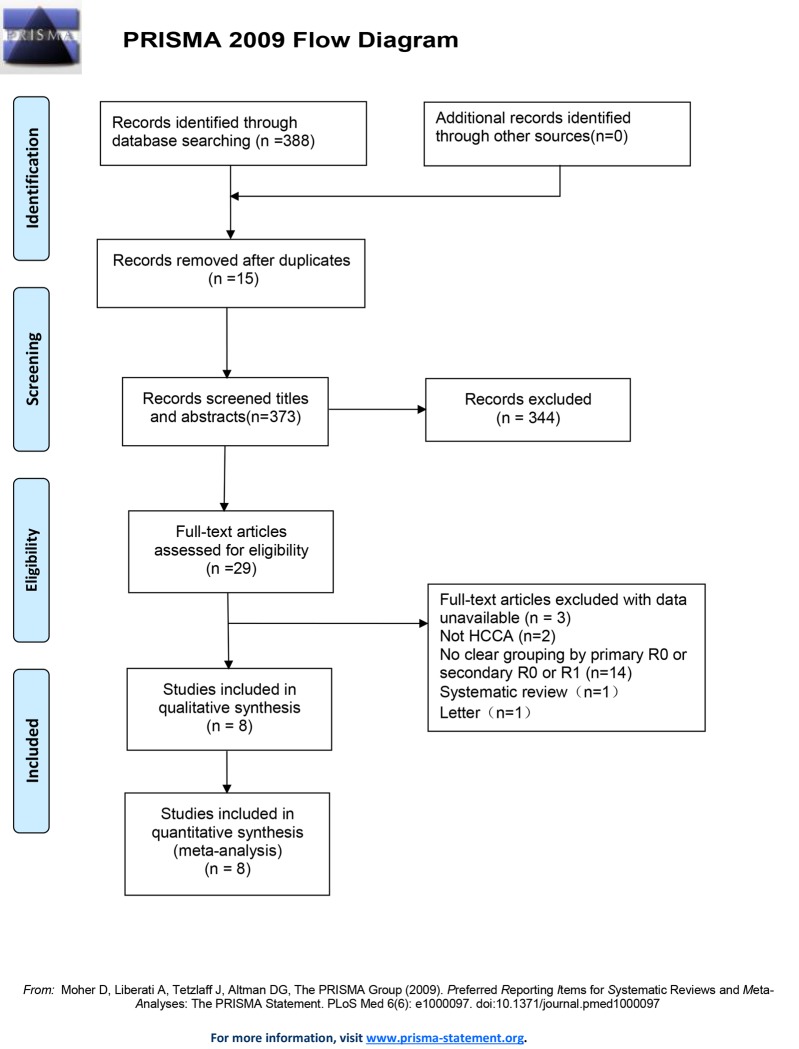
PRISMA flow diagram showing selection of articles for meta-analysis.

Initially, 388 records were identified by two independent reviewers. A total of 15 records were excluded after duplicate removal by NoteExpress 3.1. After browsing titles and abstracts, 29 records remained. And then, 19 records were excluded after full text review for the following reasons: i) two records for patients not pCCA [28, 29]; iii) 14 records for unclear grouping; ii) three records for data unavailable [21, 30, 31]. Among the remaining 10 records, one was excluded for letter [3, 32], and one for systematic review [3]. Finally, eight reports were enrolled for analysis [13, 14, 17–19, 33–35], and all of them were non-RCTs. In total, 1275 patients were enrolled in this meta-analysis, including 179 patients in the secondary R0 group, 843 patients in the primary R0 group and 253 patients in the R1 group. The search strategies and results were shown in Fig 1.

The characteristics and baseline demographic data of the patients in each research were listed in Table 1, and the surgical procedure of additional resection were depicted in Table 2.

**Table 1:** Characteristics of the clinical trials included in the meta-analysis.

Study	Center	Study years	Preoperative biliary drainage	CA19-9 (U/ml)	Bismuth type (I II III/IV)	Differentiation (well/moderate poor)	Intraoperative Frozen analysis Method	Location	Ductal resection margin status	Patients	MST (months)	Quality
Endo 2008 [34]	Memorial Sloan-Kettering Cancer Center, USA	1992–2005	79	NA	NA	29/72	NA	Proximal duct	Primary R0	54	56	7
									Secondary R0	28	38	
									R1	19	32	
Shingu 2010 [35]	Nagoya University School of Medicine, Japan	1979–2006	NA	NA	185/118	96/207	H&E	Proximal duct	Primary R0	275	NA	8
									Secondary R0	8	NA	
									R1	20	NA	
Ribero 2011 [14]	Umberto I hospital, Italy	1989–2010	NA	155(0.6–10721)	70/12	14/78	H&E	Proximal duct	Primary R0	54	29.2	8
									Secondary R0	13	30.6	
									R1	8	14.9	
Lee 2012[33]	Asan Medical Center, South korea	2000–2009	NA	NA	9/4	4/9	NA	Proximal duct	Secondary R0	7	NA	6
									R1	6	NA	
Oguro 2015 [13]	National Cancer Center Hospital, Japan	2000–2011	134	64(0–256800)	134/90	53/171	H&E	Proximal duct	Primary R0	149	56.6	8
									Secondary R0	43	29.4	
									R1	32	21.5	
Ma 2018 [17]	West China Hospital, China	2000–2017	174	195.6(5–1000)	123/105	16/212	NA	Proximal duct	Primary R0	175	23.00	8
									Secondary R0	21	20.99	
									R1	32	11.60	
Zhang 2018 [19]	Multi-center, USA	2000–2014	211	132(44.0–360.7)	177/58	44/194	NA	Proximal and distal duct	Primary R0	136	22.3	8
									Secondary R0	29	30.6	
									R1	92	18.5	
Otsuka 2019 [18]	Nagoya University Hospital, Japan	2001–2015	71	NA	NA	22/52	NA	Distal duct	Secondary R0	30	NA	7
									R1	44	NA	

NA, not available; CA19-9, carcinoma antigen 19–9; H&E, haematoxylin and eosin staining; MST, median survival time.

**Table 2:** Surgical procedures of included studies.

Study	Endo 2008 [34]	Shingu 2010 [35]	Ribero 2011 [14]	Lee 2012 [33]	Oguro 2015 [13]	Ma 2018 [17]	Zhang 2018 [19]	Otsuka 2019 [18]
Bile duct resection only	NA	15	7	30	NA	NA	56	44
Left hepatectomy	83	107	30	27	97	120	63	21
Left trisectionectomy		43	3	13	25	7	37	9
Mesohepatectomy		6	1	3	2	39	NA	NA
Right hepatectomy		100	13	65	95	49	34	33
Right trisectionectomy		12	26	19	5	13	64	8
Other hepatectomies		20	2	13	NA	NA	NA	NA
Combined caudate lobe resection	36	268	NA	NA	NA	NA	90	NA
Combined pancreatoduodenectomy	NA	28	2	3	13	NA	3	9
Combined portal vein resection	9	87	20	9	46	57	NA	20
Combined hepatic artery resection	NR	32	2	3	21	32	NA	7

NA, not available.

### Methodological quality of the included studies

The quality of each included research was shown in Table 1. Seven researches were assessed be of high quality [13, 14, 17–19, 34, 35], and one were of medium quality [33].

### Primary endpoint

**Fig 2:**
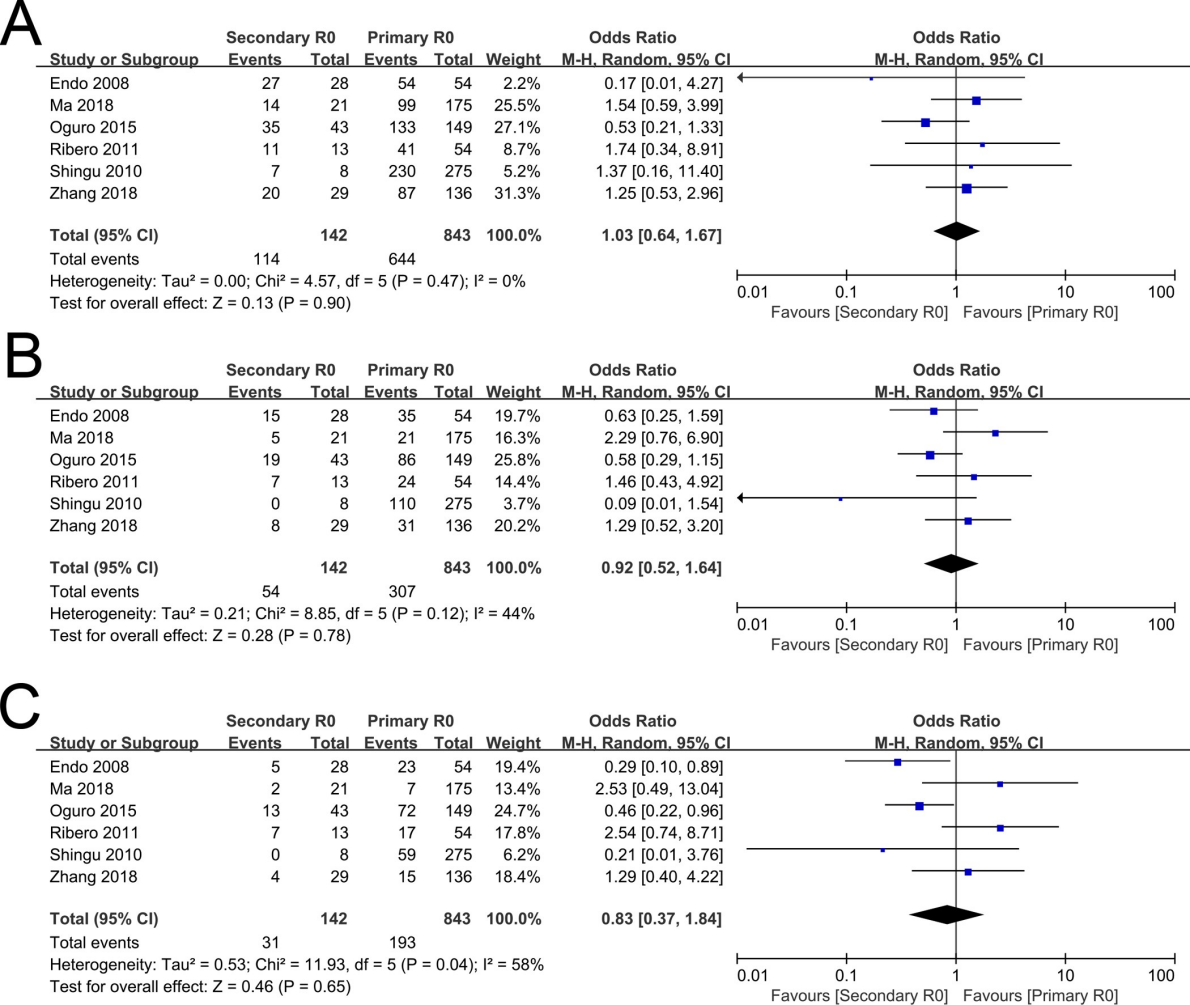
Forest plot of overall survival rates between secondary R0 group and primary R0 group. (A). 1-year overall survival rate. (B). 3-year overall survival rate. (C). 5-year overall survival rate.

The 1-, 3-, and 5-year OS rates comparing between secondary R0 group and primary R0 group were evaluated in six included studies [13, 14, 17, 19, 34, 35]. The pooled OR for the 1-, 3-, and 5-year OS rate between secondary R0 group and primary R0 group were 1.03(95%CI 0.64~1.67, P = 0.90, Fig 2A), 0.92(95%CI 0.52~1.64, P = 0.78, Fig 2B), and 0.83(95%CI 0.37~1.84, P = 0.65, Fig 2C), respectively.

**Fig 3:**
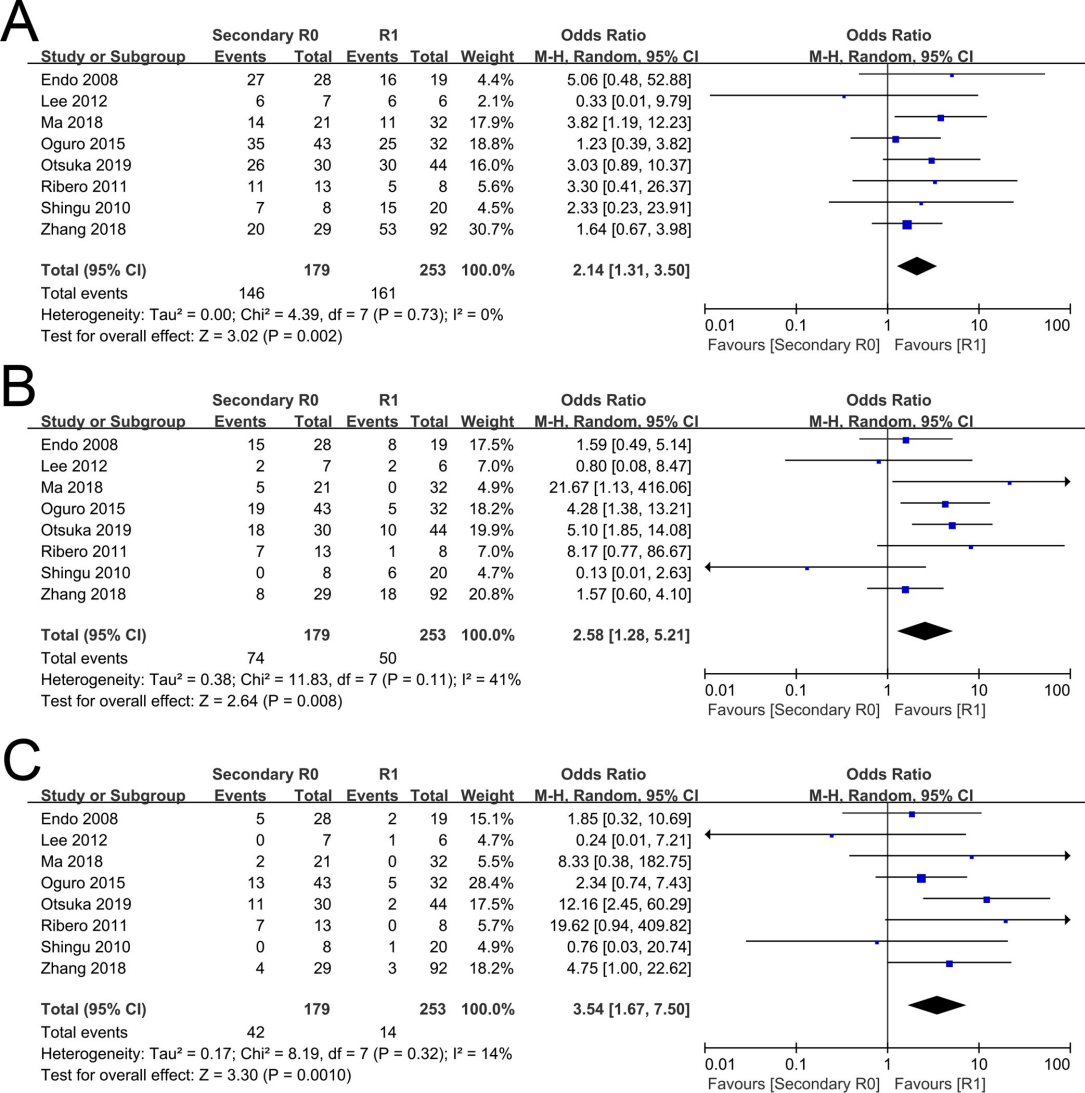
Forest plot of the overall survival rates between secondary R0 group and R1 group. (A). 1-year overall survival rate. (B). 3-year overall survival rate. (C). 5-year overall survival rate.

The 1-, 3-, and 5-year OS rates comparing between secondary R0 group and R1 group were evaluated in eight included studies [13, 14, 17–19, 33–35]. The pooled OR for the 1-, 3-, and 5-year OS rate between secondary R0 group and R1 group were 2.14(95%CI 1.31~3.50, P = 0.002, Fig 3A), 2.58(95%CI 1.28~5.21, P = 0.008, Fig 3B), and 3.54(95%CI 1.67~7.50, P = 0.001, Fig 3C), respectively.

### Subgroup analysis stratified by the West and the East

A total of three researches from the West were included in this meta-analysis [14, 19, 34], including two from the USA [19, 34] and one from Italy [14]. The pooled OR for the 1-, and 3-year OS rate between secondary R0 group and primary R0 group were 1.19(95%CI 0.58~2.44, P = 0.64, Table 3), 1.00(95%CI 0.57~1.78, P = 0.99, Table 3), respectively. And, the pooled OR for the 1-, and 3-year OS rate between secondary R0 group and R1 group were 2.05(95%CI 0.95~4.41, P = 0.07, Table 3), 1.91(95%CI 0.96~3.81, P = 0.07, Table 3), respectively.

**Table 3:** Subgroup analysis stratified by the West and the East.

Subgroup	Studies included	Overall Survival			
		Participants	Effect model	OR(95%CI)	*P*
The West, Secondary R0 vs. Primary R0					
1-year	3	314	Fixed	1.19(0.58–2.44)	0.64
3-year	3	314	Fixed	1.00(0.57–1.78)	0.99
The East, Secondary R0 vs. Primary R0					
1-year	5	671	Fixed	0.94(0.43–2.02)	0.87
3-year	5	671	Random	0.66(0.38–1.16)	0.15
The West, Secondary R0 vs. R1					
1-year	3	189	Fixed	2.05(0.95–4.41)	0.07
3-year	3	189	Fixed	1.91(0.96–3.81)	0.07
The East, Secondary R0 vs. R1					
1-year	5	243	Fixed	2.19(1.18–4.07)	**0.01**
3-year	5	243	Fixed	3.21(1.75–5.89)	**<0.001**

A total of five researches from the East were included in this meta-analysis [13, 17, 18, 33, 35], including three from Japan [13, 18, 35], one from South Korea [33], and one from China [17]. The pooled OR for the 1-, and 3-year OS rate between secondary R0 group and primary R0 group were 0.94(95%CI 0.43~2.02, P = 0.87, Table 3), 0.66(95%CI 0.38~1.16, P = 0.15, Table 3), respectively. And, the pooled OR for the 1-, and 3-year OS rate between secondary R0 group and R1 group were 2.19(95%CI 1.18~4.07, P = 0.01, Table 3), 3.21(95%CI 1.75~5.89, P<0.001, Table 3), respectively.

### Prognostic factors for pCCA after resection

Prognostic factors were analyzed in six studies using univariate and multivariate analysis [13, 17, 19, 33–35]. Tumor differentiation, lymph node involvement, combined PV and/or HA, microscopic venous invasion, microscopic liver invasion, and tumor stage were confirmed to be independent risk factors of overall survival. Details were depicted in Table 4.

**Table 4:** Meta-analysis of prognostic factors for pCCA after resection.

Prognostic factors	Studies included	Heterogeneity		Hazard ratio	95%CI
		*I*^*2*^ *(%)*	*P*		
Tumour differentiation (others versus well differentiated)	6	0	0.51	2.25	1.82–2.79
Lymph node involvement (yes versus no)	6	0	0.83	1.85	1.55–2.21
Combined PV and/or HA (yes versus no)	3	37	0.21	1.37	1.11–1.69
Microscopic perineural invasion (yes versus no)	3	66	0.05	1.59	0.94–2.68
Microscopic venous invasion (yes versus no)	2	0	0.98	1.42	1.08–1.86
Microscopic liver invasion (yes versus no)	2	0	0.46	1.43	1.08–1.90
Tumour status (T3+T4 versus T1+T2)*	2	0	0.43	1.23	1.02–1.46

PA, portal vein resection; HA, hepatic artery resection;

*according to the 8^th^ edition American Joint Committee on Cancer (AJCC) staging guidelines.

### Sensitivity analysis and publication bias

**Fig 4:**
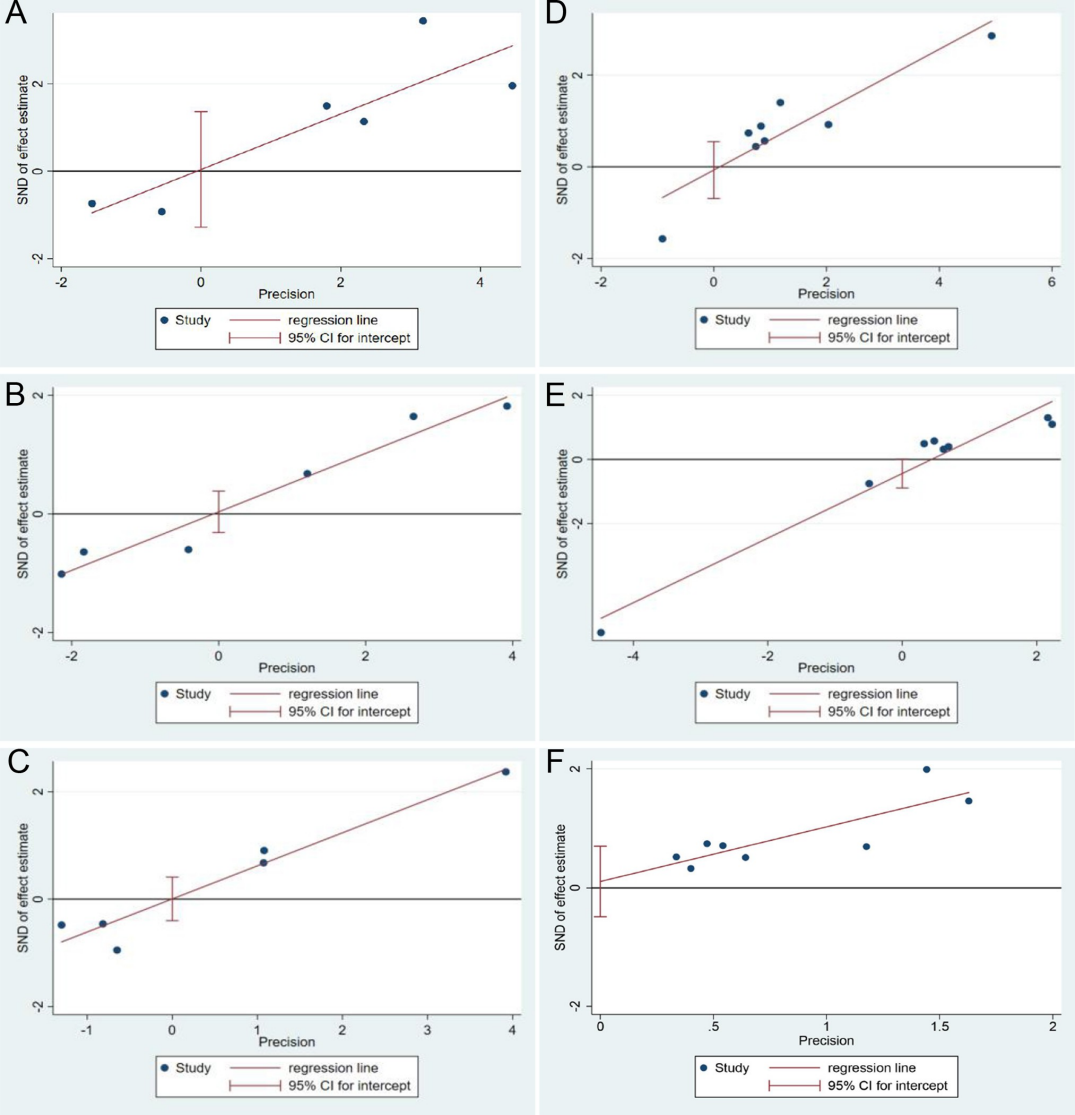
Publication bias in this study. (A). 1-year overall survival rate between secondary R0 group and primary R0 group. (B). 3-year overall survival rate between secondary R0 group and primary R0 group. (C.) 5-year overall survival rate between secondary R0 group and primary R0 group. (D). 1-year overall survival rate between secondary R0 group and R1 group. (E). 3-year overall survival rate between secondary R0 group and R1 group. (F). 5-year overall survival rate between secondary R0 group and R1 group.

Removal of any individual study did not affect the overall outcome of this meta-analysis significantly in the sensitivity analysis. Significant publication bias was not observed in the pooled OR for 1-, 3-, and 5-year OS between secondary R0 and primary R0/R1 (all P >0.05, Fig 4).

## Discussion

This was the first meta-analysis that evaluated the clinical value of additional resection of intraoperative R1 for pCCA. A total of 8 studies with 1275 patients were included in this meta-analysis. Results showed that the 1-, 3-, and 5-year OS rates were comparable between patients with secondary R0 and patients with primary R0, but both are better than patients with R1. Hence, additional resection for pCCA was recommended in case of intraoperative R1 to achieve a better prognosis, especially in selected patients.

R0 resection is a standard procedure for curative pCCA, although the detailed procedures are different from each other worldwide [4, 36–38]. But the incidence of R1 was as high as 10~72% [11, 13, 14], mainly due to the complex anatomy of hepatic hilar and biological characteristics of pCCA. And, patients with R1 had a poor prognosis [11–13]. In this meta-analysis, the initial R1 ranged from 12.3% to 37.2%. Hence, additional resection of intraoperative pathologic R1 for pCCA is necessary to achieve a better prognosis. However, it remains controversial whether additional resection could benefit the patients with intraoperative R1 in the view of long-term prognosis [13, 14, 17, 19]. In this meta-analysis, we found that the 1-, 3-, and 5-year OS rates in the secondary R0 group were higher than those in the R1 group, but the median survival time (MST) in the primary R0, secondary R0, and R1 were 22.3–56.6 months, 20.99–38 months, and 11.60–32 months, respectively. Hence, the conclusion that patients with intraoperative pathologic R1 would be benefited from additional resection needed further validation.

However, not all patients with intraoperative R1 would be benefited from resection. Major differences were found in patient characteristics and treatment strategies between the East and the West pCCA cohorts [39]. In the subgroup of the West, there were no significant difference in the pooled OR for the 1- and 3- year OS of patients between secondary R0 group and R1 group, which suggested that additional resection would not benefit patients in the West. Reasons might be as follows: 1) epidemiology of pCCA was greatly different between the West and East, the incidence of pCCA was much higher in the East than that in the West [40]. What’s more, liver fluke was the leading etiology of pCCA in the East while it was primary sclerosing cholangitis in the West [40]; 2) cirrhosis was an important factor of decision-making on hepatectomy, which was much more frequent and serious in the East [39]; 3) pCCA patients were typically present with obstructive jaundice, and endoscopic biliary drainage was found to be associated with improved prognosis compared with percutaneous biliary drainage, which was conducted much more frequently in the East [41]; 4) preoperative portal vein embolization was repeatedly confirmed to be able to improve the prognosis of pCCA via increasing the future remnant liver volume, which was also performed much often in the East [42, 43]; 4) surgical techniques including vascular reconstruction, and lymph node dissection have been conducted prevalently in Japan, South Korea, and China without increased risk of severe postoperative complications [39].

In an attempt to achieve a secondary R0, more extensive resection would be proposed, which would increase the risk of morbidity and mortality in turn. Liver failure, biliary fistula, anastomotic leak, surgical site infection, intra-abdominal bleeding, and vascular complications are the common complications related to additional resection, and the morbidity ranged from 40~71.2% [19, 44]. The procedure related postoperative mortality varied from 2 to 15% [45, 46], although it was reported to be decreased [9]. Additional resection should better be conducted in highly experienced centers if future remnant liver volume was adequate and additional resection was feasible in anatomy, but data on morbidity and mortality was unavailable in most of the included studies. In addition, considering that the longitudinal infiltration of pCCA along the bile duct was confirmed to be 4.6mm~14.0mm [47], minor additional resection was recommended in case of intraoperative R1, on condition that a secondary R0 was guaranteed.

As is known to all, one size does not fit for all. R1 typical includes R-inv and R-cis, and R-inv is much more aggressive than R-cis in pathology theoretically [3, 29]. However, recent studies showed that addition resection could not improve the prognosis of extrahepatic cholangiocarcinoma patients with initial R-cis [18, 48]. In our newly published meta-analysis, no significant differences were observed in the 1-, 2-, and 3-year survival rates between groups of R0 and R-cis (all P>0.05), both of which were significantly higher than those in the group of R-inv (all P<0.05) [49], which indicated that additional resection would be not necessary for patients with initial R-cis. Unfortunately, relevant data was unable to extract in the included studies, and further studies are badly needed on this issue.

Intraoperative frozen section analysis is prevalently used to assess resection margins during cancer surgery, and is a determinant whether addition resection is warranted to achieve R0 [50, 51]. But the clinical value of intraoperative frozen section in pCCA was limited, because the sensitivity was reported to be only 68% [21]. The inconsistency between frozen section and permanent histopathological analyses of bile ductal margin might be explained by as follows: 1) the biological characteristics of pCCA, because pCCA often spread longitudinally along the axis of the bile duct, partially in the submucosal space with wide invasion to the perilymph, perineural and perivasculature [52], which increased the risk of sampling errors; 2) the presence of atypical cells within the boundary zone between the tumor and the normal duct epithelium [34]; 3) unrecognized margin due to energy surgical instruments such as CUSA, Endo-GIA and so on. However, intraoperative R1 could put the surgeons into a dilemma because additional resection increased the risk of morbidity or was sometimes impossible anatomically.

There were several restrictions of this meta-analysis. First, all the included studies were retrospective studies, indicating an apparent recalling bias and selection bias. Secondly, major differences were found in pCCA between the West and the East, and the results of subgroup analysis were indeed different. Thirdly, the procedures of radical resection for pCCA were different from different countries [13, 14, 17, 19]. Fourth, the safety of addition resection was not evaluated because postoperative morbidity and mortality was only reported in one study. Fifth, right or left hepatectomy was depended on the Bismuth-Corlette type, which would be affected the prognosis of pCCA, but the data was unavailable. Sixth, margin status including either R-inv or R-cis was the key, but relevant subgroup analysis was unable to conduct due to insufficient data. The last but not the least, publication bias was hard to be avoided, although significant publication bias was not observed in the study.

## Conclusion

With the current data, we concluded that additional resection should be recommended in selected patients with intraoperative R1, especially in highly experienced centers. However, the standard procedure of radical resection for pCCA and a higher sensitivity and specificity margin examining are badly needed in clinical. In addition, further studies concerning on the types of R1 are also needed.

## Supporting information

S1 Table Checklist_PRISMA. (DOC)

S2 Table Search strategy in PubMed. (DOCX)

## Abbreviations

HCCA: hilar cholangiocarcinoma
pCCA: perihilar cholangiocarcinoma
OS: overall survival
OR: Odds ratio
CI: confidence interval
RCT: randomized controlled trial
PA: portal vein resection
HA: hepatic artery resection
PM: proximal bile duct margin
DM: distal bile duct margin
