# Supplementary material for: Does additional resection of a positive microscopic ductal margin benefit patients with perihilar cholangiocarcinoma: A systematic review and meta-analysis
Source: PLoS One. 2020 May 7;15(5):e0232590. doi: 10.1371/journal.pone.0232590 (PMC7205232; doi:10.1371/journal.pone.0232590)
Supplement: S2 Table — (DOCX) [file pone.0232590.s002.docx]

**Search strategy in PubMed**

| #1 “hilar cholangiocarcinoma”[MeSH Terms] OR “hilar cholangiocarcinoma”[All Fields] OR “perihilar cholangiocarcinoma”[MeSH Terms] OR “perihilar cholangiocarcinoma”[All Fields] OR “Klatskin’s tumor”[MeSH Terms] OR “Klatskin’s tumor”[All Fields] |
| --- |
| #2 “additional resection”[MeSH Terms] OR “additional resection”[All Fields] OR “extensive resection”[MeSH Terms] OR “extensive resection”[All Fields] OR “re-resection”[MeSH Terms] OR “re-resection”[All Fields] |
| #3 “margin”[MeSH Terms] OR “margin”[All Fields] |
| #4 #1 and #2 and #3 |
